# The Unbearable Weight of Autonomy: Simulating Moral Distress and Fundamental Legal Concepts for Psychiatry Trainees

**DOI:** 10.1192/bjo.2026.11937

**Published:** 2026-06-30

**Authors:** Adam Flynn

**Affiliations:** Northern Ireland Medical & Dental Training Agency, Belfast, United Kingdom

## Abstract

**Aims:**

In Northern Ireland, the prevalence of mental health disorders is estimated to be significantly higher than in England, set against a backdrop of socioeconomic deprivation. Consequently, a resilient workforce is a clinical imperative. However, postgraduate training often leaves a critical gap between theoretical knowledge of The Mental Health Order (NI) 1986 and the emotional endurance required to apply its principles in acute settings. This paper describes the development of a high-fidelity simulation designed to bridge this gap. The primary aim was to transform a morally injurious personal experience into a safe educational tool, teaching trainees to navigate the conflict between the Hippocratic instinct to treat and the strict statutory limits regarding patient autonomy.

**Methods:**

A high-fidelity simulation was developed within the Western Health & Social Care Trust as part of a Psychiatric Emergencies Simulation Day. Grounded in Kolb’s experiential learning cycle and the author’s dual background as a Solicitor and Psychiatrist, the scenario involves a voluntary patient with Emotionally Unstable Personality Disorder (EUPD) and Type 1 Diabetes refusing insulin. The patient is fully capacitous, aware of the risk of Diabetic Ketoacidosis (DKA), yet refuses treatment due to emotional dysregulation. The simulation utilizes Bion’s theory of containment; the facilitator absorbs trainee anxiety (counter-transference) to prevent the loss of a mentalizing stance, modelling how to regulate the urge to utilize coercive measures prematurely.

**Results:**

The simulation exposes the visceral conflict between the right to life (ECHR Article 2) and the right to private life/autonomy (ECHR Article 8). Trainees are forced to confront the specific constraints of the MHO (NI) 1986, which precludes detention solely based on Personality Disorder. The scenario demonstrates that the threshold for overriding a competent refusal is the high bar of the Common Law doctrine of Necessity. The primary learning outcome is the realization that the hardest clinical decision is often not to treat.

**Conclusion:**

This educational intervention successfully establishes that respecting legally protected autonomy, even when it risks catastrophe, exacts a heavy toll of moral distress. By integrating legal jurisprudence with psychotherapeutic principles of containment, the simulation acts as a sublimation of moral injury. It demonstrates that curriculum development must address the “faceless” demands of the law alongside the human needs of the doctor. Providing a controlled environment to bear the “unbearable weight of autonomy” is an ethical imperative for mitigating burnout and ensuring true patient-centred care.

